# Complete genome sequences of *Bifidobacterium faecale* strain JCM19861^T^, isolated from human feces

**DOI:** 10.1128/mra.00784-23

**Published:** 2023-12-15

**Authors:** Shinnosuke Okuhama, Hidenori Takahashi, Yasunori Nakayama, Yusuke Ogata, Wataru Suda

**Affiliations:** 1 Biolier Business Department, Teijin Limited, Tokyo, Japan; 2 Laboratory for Microbiome Sciences, RIKEN Center for Integrative Medical Sciences, Yokohama, Japan; 3 Laboratory for Symbiotic Microbiome Sciences, RIKEN Center for Integrative Medical Sciences, Yokohama, Japan; University of Maryland School of Medicine, Baltimore, Maryland, USA

**Keywords:** *B. faecale* JCM19861T, complete genome sequences

## Abstract

Here, we report the complete genome sequence of the *Bifidobacterium faecale* strain JCM 19861^T^ (= CU 3-7^T^ = KACC 17904^T^), isolated from infant feces by Jung-Hye Choi’s group in 2014. The *B. faecale* JCM 19861^T^ genome comprised a circular chromosome of 2,213,206 bp, with a G + C content of 59.0%.

## ANNOUNCEMENT

The *Bifidobacterium* was discovered by Tisser (1900) in the feces of infants as *Bacillus bifidus*, a rod-shaped, gram-positive, anaerobic bacterium, and bifid morphology and reclassified by Orla-Jensen (1924) as a separate taxon (1). Some species of this genus are available to humans and animals as probiotic foods for health worldwide (2). *Bifidobacterium faecale* strain JCM19861^T^ (= CU 3–7^T^ = KACC 17904^T^) was isolated from the feces of a 2-week-old baby as a type strain of a novel species by Jung-Hye Choi’s group. 16S rRNA gene sequence similarity of strain JCM19861^T^ (Accession No. KF990498.1) with *B. adolescentis* KCTC 3216^T^ and *B. ruminantium* KCTC 3425^T^ showed, respectively, 98.4% and 97.9% (3).

*B. faecale* JCM 19861^T^ was provided by the RIKEN BRC through the National BioResource Project of the MEXT, Japan. The strain JCM 19861^T^ was grown under anaerobic conditions in Gifu Anaerobic Medium (Nissui Pharmaceutical Co., Ltd., Japan) with 0.7% glucose for 48 h. The bacterial pellet of *B. feacale* JCM 19861^T^ was incubated with 15 mg/mL lysozyme and then treated with 1 mg/mL proteinase K and 1% sodium dodecyl sulfate (4). After that, the genomic DNA was extracted with phenol-chloroform-isoamyl alcohol, isolated by ethanol precipitation, and purified by RNase A treatment, followed by polyethylene glycol 6000 precipitation (5). Whole-genome sequencing was performed using a PacBio Sequel II system [Pacific Biosciences of California, Inc., USA (PacBio)]. The library was prepared with SMRTbell express template preparation kit 2.0 (PacBio) with DNA shearing by gTUBE (Covaris, LLC, USA) and with a 10- to 15-kb target length. In the process after the PacBio reads collection, default parameters were used for all software unless otherwise specified. The PacBio reads were converted to circular consensus sequencing (HiFi) reads using the ccs software (https://github.com/PacificBiosciences/ccs; v.6.2.0) and assembled using the assembler Canu (v.2.2) with specified parameters (minReadLength  =  2,200; minOverlapLength  =  2,200) (6). The HiFi reads remapped to the generated contigs by Minimap2 (v2.24-r1122) (7) and those contigs with <5× coverage were removed. Then, the generated circular contig was checked for circularization to remap the HiFi reads and the linear contigs these mapped to the circular chromosome with ≥99% identity and ≥95% coverage were removed as bubbles. The obtained genomic sequence was annotated using the DDBJ Fast Annotation and Submission Tool (DFAST v1.2.18, https://dfast.nig.ac.jp) (8).

We obtained a total of 76,936,871 bases and 6,288 Sequel reads with an N50 value of 12,006 bp and the reads assembly generated a circular contig with a 35× coverage, corresponding to the *B. faecale* strain JCM 19861^T^ chromosome.

The *B. faecale* JCM 19861^T^ genome comprised a circular chromosome of 2,213,206 bp, with a G + C content of 59.0%, containing 1,884 protein-coding genes with 58 tRNA, 13 rRNA, and 1 CRISPR genes.

## Data Availability

The complete genome sequence of *B. faecale* JCM 19861^T^ was deposited in DDBJ/ENA/GenBank under accession no. AP028457, which is linked to the BioProject accession no. PRJDB16183, the BioSample accession no. SAMD00630223, and the DDBJ Sequence Read Archive (DRA) accession no. DRR491367.
